# Supplementary figures and images for: An online survey of informal caregivers’ unmet needs and associated factors
Source: PLoS One. 2020 Dec 10;15(12):e0243502. doi: 10.1371/journal.pone.0243502 (PMC7728235; doi:10.1371/journal.pone.0243502)

**S1 Table. Unmet needs survey**


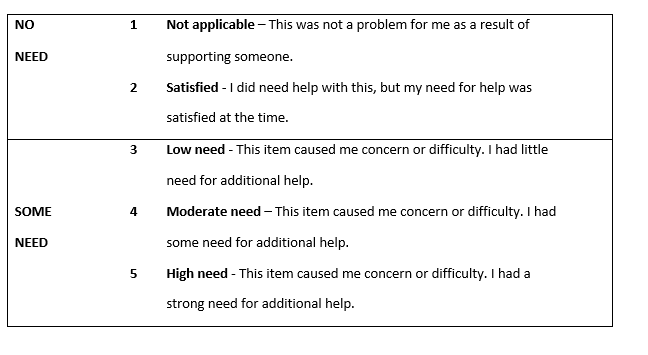

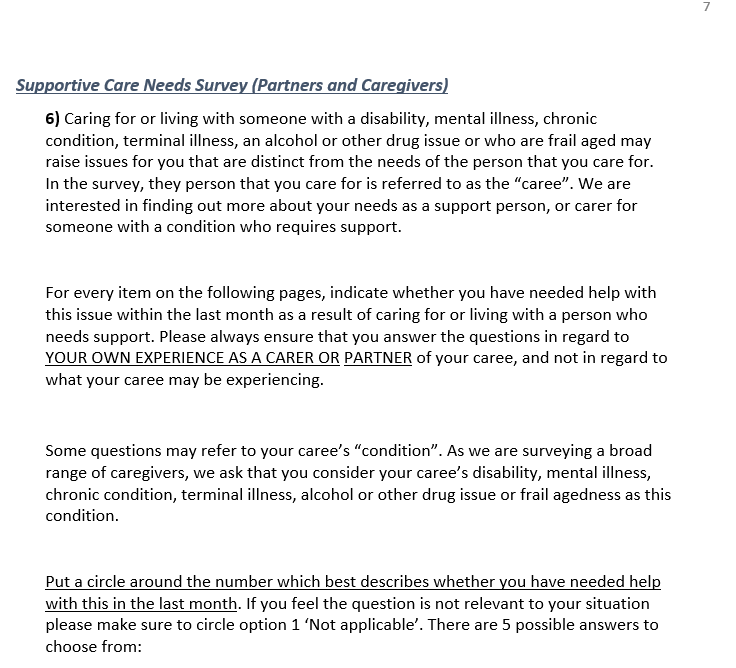


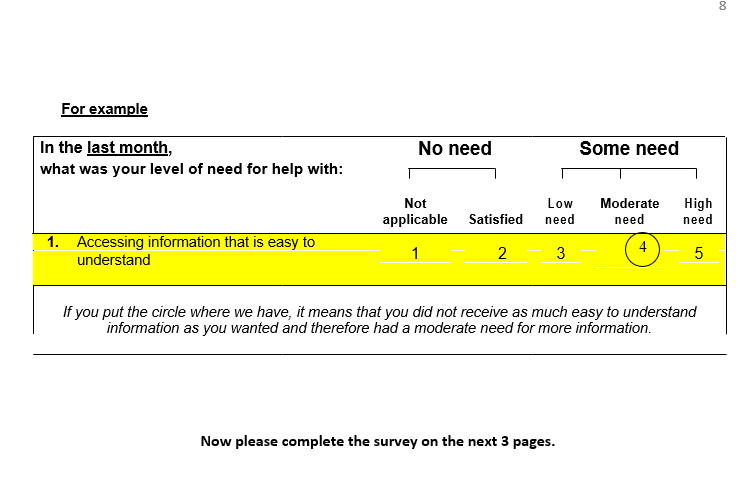


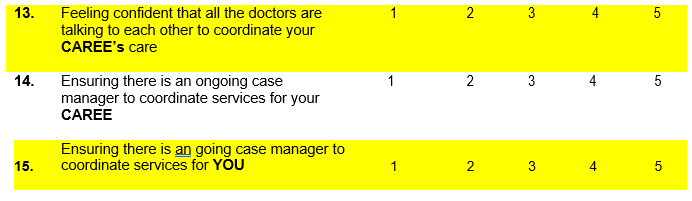

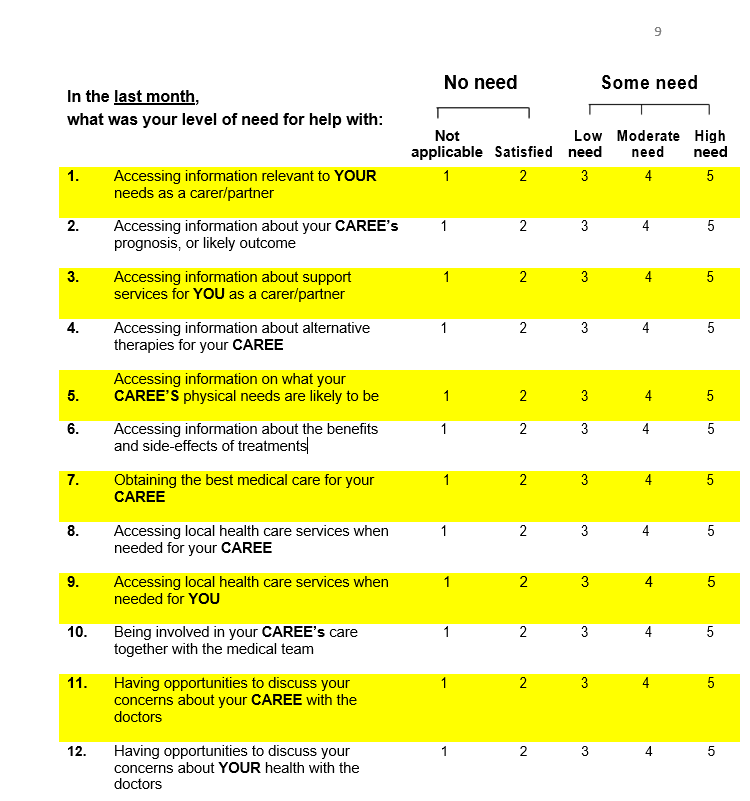


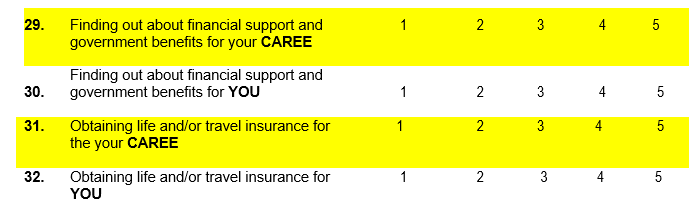

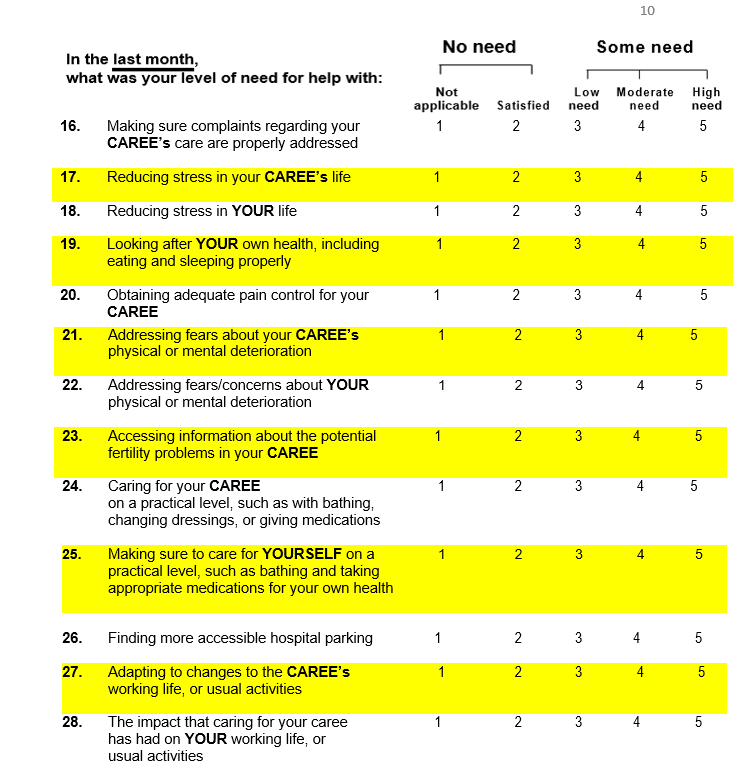


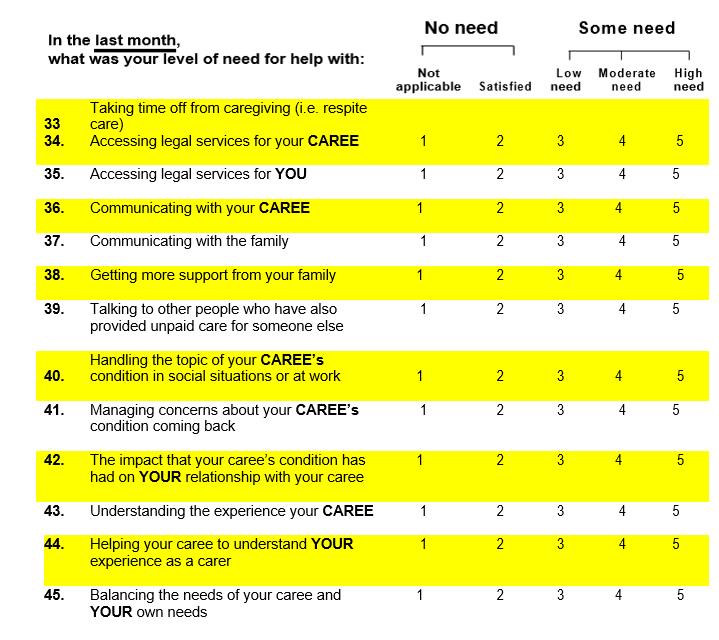

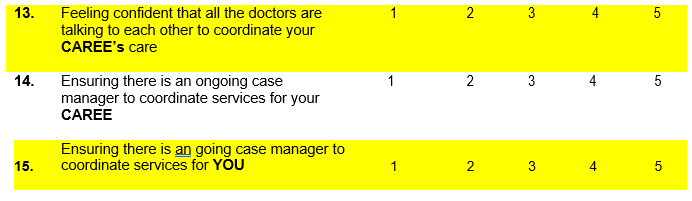

Supplement: S1 Table — Modified unmet needs survey based on the Supportive Care Needs Survey-Partners and Caregivers (SCNS-P&C). (DOCX) [file pone.0243502.s001.docx]
