# Supplementary material for: An online survey of informal caregivers’ unmet needs and associated factors
Source: PLoS One. 2020 Dec 10;15(12):e0243502. doi: 10.1371/journal.pone.0243502 (PMC7728235; doi:10.1371/journal.pone.0243502)
Supplement: S2 Table — Factor analysis used to establish the types of unmet needs reported by caregivers. (DOCX) [file pone.0243502.s002.docx]

**S2 Table. Factor analysis**

| **Item** | **Variable / Item Name** | **Health Information and Support for Care Recipient** | **Health Service Management** | **Communication and Relationship** | **Self-care** | **Support Services Accessibility** |
| --- | --- | --- | --- | --- | --- | --- |
| 1 | Accessing information relevant to YOUR needs as a carer/partner | 0.43 |  |  | **0.44**** |  |
| 2 | Accessing information about your CAREE’s prognosis, or likely outcome | **0.76**** |  |  |  |  |
| 3 | Accessing information about support services for YOU as a carer/partner | 0.39 |  |  | **0.52**** |  |
| 4 | Accessing information about alternative therapies for your CAREE | **0.66**** |  |  |  |  |
| 5 | Accessing information on what your CAREE’S physical needs are likely to be | **0.68**** |  |  |  |  |
| 6 | Accessing information about the benefits  and side-effects of treatments | **0.77**** |  |  |  |  |
| 7 | Obtaining the best medical care for your CAREE | **0.81**** |  |  |  |  |
| 8 | Accessing local health care services when needed for your CAREE | **0.73**** |  |  |  |  |
| 9 | Accessing local health care services when needed for YOU | 0.40 |  |  | **0.63**** |  |
| 10 | Being involved in your CAREE’s care together with the medical team | **0.77**** |  |  |  |  |
| 11 | Having opportunities to discuss your concerns about your CAREE with the doctors | **0.74**** |  |  |  |  |
| 12 | Having opportunities to discuss your concerns about YOUR health with the doctors | 0.41 |  |  | **0.59**** |  |
| 13 | Feeling confident that all the doctors are talking to each other to coordinate your CAREE’s care | 0.31 | **0.54**** |  |  |  |
| 14 | Ensuring there is an ongoing case  manager to coordinate services for your CAREE |  | **0.85**** |  |  |  |
| 15 | Ensuring there is an going case manager to coordinate services for YOU |  | 0.62 |  | **0.43**** |  |
| 16 | Making sure complaints regarding your CAREE’s care are properly addressed |  | **0.56**** |  |  |  |
| 17 | Reducing stress in your CAREE’s life |  | **0.50**** |  |  |  |
| 18 | Reducing stress in YOUR life |  |  | 0.34 | **0.57**** |  |
| 19 | Looking after YOUR own health, including eating and sleeping properly |  |  |  | **0.63**** |  |
| 20 | Obtaining adequate pain control for your CAREE |  | **0.37**** |  |  |  |
| 21 | Addressing fears about your CAREE’s physical or mental deterioration | 0.43 |  | **0.36**** |  |  |
| 22 | Addressing fears/concerns about YOUR physical or mental deterioration |  |  |  | **0.65**** |  |
| 23 | Accessing information about the potential fertility problems in your CAREE |  |  |  |  | **0.61**** |
| 24 | Caring for your CAREE  on a practical level, such as with bathing, changing dressings, or giving medications |  | **0.41**** |  |  |  |
| 25 | Making sure to care for YOURSELF on a practical level, such as bathing and taking appropriate medications for your own health |  |  |  |  | ** |
| 26 | Finding more accessible hospital parking |  | **0.32**** |  |  |  |
| 27 | Adapting to changes to the CAREE’s  working life, or usual activities |  |  | **0.31**** |  |  |
| 28 | The impact that caring for your caree has had on YOUR working life, or usual activities |  |  | 0.41 | **0.41**** |  |
| 29 | Finding out about financial support and government benefits for your CAREE |  | 0.37 |  |  | ** |
| 30 | Finding out about financial support and government benefits for YOU |  |  |  | **0.40**** | 0.37 |
| 31 | Obtaining life and/or travel insurance for the your CAREE |  |  |  |  | **0.83**** |
| 32 | Obtaining life and/or travel insurance for YOU |  |  |  |  | **0.84**** |
| 33 | Taking time off from caregiving (i.e. respite care) |  |  | 0.36 | **0.42**** |  |
| 34 | Accessing legal services for your CAREE |  | 0.41 |  |  | **0.54**** |
| 35 | Accessing legal services for YOU |  |  |  |  | **0.62**** |
| 36 | Communicating with your CAREE |  |  | **0.55**** |  |  |
| 37 | Communicating with the family |  |  | **0.55**** |  |  |
| 38 | Getting more support from your family |  |  | **0.61**** |  |  |
| 39 | Talking to other people who have also provided unpaid care for someone else |  |  | **0.56**** |  |  |
| 40 | Handling the topic of your CAREE’s condition in social situations or at work |  |  | **0.74**** |  |  |
| 41 | Managing concerns about your CAREE’s condition coming back |  |  | **0.47**** |  |  |
| 42 | The impact that your caree’s condition has had on YOUR relationship with your caree |  |  | **0.77**** |  |  |
| 43 | Understanding the experience your CAREE |  |  | **0.59**** |  |  |
| 44 | Helping your caree to understand YOUR experience as a carer |  |  | **0.71**** |  |  |
| 45 | Balancing the needs of your caree and YOUR own needs |  |  | **0.73**** | 0.34 |  |
| **Denotes the factor loading which was used to determine which factor the item best aligned with | | | | | | |
