# Supplementary material for: An online survey of informal caregivers’ unmet needs and associated factors
Source: PLoS One. 2020 Dec 10;15(12):e0243502. doi: 10.1371/journal.pone.0243502 (PMC7728235; doi:10.1371/journal.pone.0243502)
Supplement: S3 Table — (DOCX) [file pone.0243502.s003.docx]

**S3 Table. Ranked highest moderate or high unmet needs reported in the last month by informal caregivers**

| **Rank** | | **Variable / Item Name** | | **% of sample reporting a moderate-high need** | | **Domain** | |
| --- | --- | --- | --- | --- | --- | --- | --- |
| 1 | | Reducing stress in YOUR life | | 74.5 | | Self-care | |
| 2 | | Balancing the needs of your caree and YOUR own needs | | 71.3 | | Communication and relationship | |
| 3 | | Looking after YOUR own health, including eating and sleeping properly | | 71.2 | | Self-care | |
| 4 | | The impact that caring for your caree has had on YOUR working life, or usual activities | | 70.6 | | Self-care | |
| 5 | | Taking time off from caregiving (i.e. respite care) | | 62.6 | | Self-care | |
| 6 | | Accessing information about support services for YOU as a carer/partner | | 59.7 | | Self-care | |
| 7 | | Ensuring there is an ongoing case manager to coordinate services for your CAREE | | 54.3 | | Health service management | |
| 8 | | Helping your caree to understand YOUR experience as a carer | | 51.2 | | Communication and relationship | |
| 9 | | Accessing information relevant to YOUR needs as a carer/partner | | 51.0 | | Self-care | |
| 10 | | Addressing fears/concerns about YOUR physical or mental deterioration | | 51.0 | | Self-care | |
| 11 | | Finding out about financial support and government benefits for your CAREE | | 51.0 | | Support services accessibility | |
| 12 | | Finding out about financial support and government benefits for YOU | | 50.7 | | Self-care | |
| 13 | | The impact that your caree’s condition has had on YOUR relationship with your caree | | 50.1 | | Communication and relationship | |
| 14 | | Feeling confident that all the doctors are talking to each other to coordinate your CAREE’s care | | 49.3 | | Health service management | |
| 15 | | Addressing fears about your CAREE’s physical or mental deterioration | | 49.3 | | Communication and relationship | |
| 16 | | Reducing stress in your CAREE’s life | | 49.0 | | Health service management | |
| 17 | | Ensuring there is an going case manager to coordinate services for YOU | | 48.3 | | Self-care | |
| 18 | | Getting more support from your family | | 47.4 | | Communication and relationship | |
| 19 | | Accessing information about alternative therapies for your CAREE | | 46.9 | | Health information and support for care recipient | |
| 20 | | Obtaining the best medical care for your CAREE | | 46.9 | | Health information and support for care recipient | |
| 21 | | Having opportunities to discuss your concerns about YOUR health with the doctors | | 45.1 | | Self-care | |
| 22 | | Accessing local health care services when needed for your CAREE | | 43.9 | | Health information and support for care recipient | |
| 23 | | Accessing local health care services when needed for YOU | | 43.5 | | Self-care | |
| 24 | | Having opportunities to discuss your concerns about your CAREE with the doctors | | 42.5 | | Health information and support for care recipient | |
| 25 | | Accessing information about your CAREE’s prognosis, or likely outcome | | 41.0 | | Health information and support for care recipient | |
| 26 | | Accessing information about the benefits and side-effects of treatments | | 39.3 | | Health information and support for care recipient | |
| 27 | | Accessing information on what your CAREE’S physical needs are likely to be | | 38.9 | | Health information and support for care recipient | |
| 28 | | Making sure complaints regarding your CAREE’s care are properly addressed | | 38.7 | | Health information and support for care recipient | |
| 29 | | Talking to other people who have also provided unpaid care for someone else | | 37.6 | | Communication and relationship | |
| 30 | | Adapting to changes to the CAREE’s working life, or usual activities | | 36.8 | | Communication and relationship | |
| 31 | | Being involved in your CAREE’s care together with the medical team | | 34.8 | | Health information and support for care recipient | |
| 32 | | Understanding the experience your CAREE | | 33.1 | | Communication and relationship | |
| 33 | | Handling the topic of your CAREE’s condition in social situations or at work | | 32.5 | | Communication and relationship | |
| 34 | | Finding more accessible hospital parking | | 31.0 | | Health service management | |
| 35 | | Communicating with the family | | 29.1 | | Communication and relationship | |
| 36 | | Managing concerns about your CAREE’s condition coming back | | 28.4 | | Communication and relationship | |
| 37 | | Communicating with your CAREE | | 27.9 | | Communication and relationship | |
| 38 | | Caring for your CAREE on a practical level, such as with bathing, changing dressings, or giving medications | | 27.3 | | Health service management | |
| 39 | | Obtaining adequate pain control for your CAREE | | 25.0 | | Health service management | |
| 40 | | Accessing legal services for your CAREE | | 24.7 | | Support services accessibility | |
| 41 | | Accessing legal services for YOU | | 21.4 | | Support services accessibility | |
| 42 | | Obtaining life and/or travel insurance for the your CAREE | | 15.9 | | Support services accessibility | |
| 43 | | Making sure to care for YOURSELF on a practical level, such as bathing and taking appropriate medications for your own health | | 12.9 | | Support services accessibility | |
| 44 | | Obtaining life and/or travel insurance for YOU | | 12.9 | | Health service management | |
| 45 | | Accessing information about the potential fertility problems in your CAREE | | 5.7 | | Support services accessibility | |
