# Supplementary material for: An online survey of informal caregivers’ unmet needs and associated factors
Source: PLoS One. 2020 Dec 10;15(12):e0243502. doi: 10.1371/journal.pone.0243502 (PMC7728235; doi:10.1371/journal.pone.0243502)
Supplement: S4 Table — (DOCX) [file pone.0243502.s004.docx]

**S4 Table. The crude self-reported socioeconomic, disease-related and country related variable associated with reporting moderate-high unmet need in each factor domain; logistic regression model**

|  | Health Information and Support for Care Recipient | | | Health Service Management | | | Support Services Accessibility | |
| --- | --- | --- | --- | --- | --- | --- | --- | --- |
|  | | Odds Ratio (CI) | *p* | Odds Ratio (CI) | *p* | Odds Ratio (CI) | | *p* |
| Age (years) | |  | 0.019 |  | 0.004 |  | | <0.001 |
| 18 - 45 | | 2.81 (1.33, 5.93) | 0.007 | 3.51 (1.67, 7.36) | 0.001 | 3.90 (2.06, 7.40) | | <0.001 |
| 45 – 65 | | 1.27 (0.73, 2.20) | 0.396 | 1.66 (0.97, 2.89) | 0.064 | 2.50 (1.48, 4.21) | | 0.001 |
| 65+ | | 1 |  | 1 |  | 1 | |  |
| Gender | |  |  |  |  |  | |  |
| Female | | 1 |  | 1 |  | 1 | |  |
| Male | | 1.21 (0.64, 2.27) | 0.559 | 0.93 (0.48, 1.79) | 0.818 | 1.69 (0.96, 2.97) | | 0.068 |
| Country | |  | 0.800 |  | 0.615 |  | | 0.171 |
| Australia | | 1 |  | 1 |  | 1 | |  |
| Canada | | 1.13 (0.57, 2.21) | 0.730 | 0.90 (0.45, 1.80) | 0.766 | 0.78 (0.43, 1.42) | | 0.418 |
| New Zealand | | 1.15 (0.51, 2.56) | 0.740 | 0.55 (0.26, 1.16) | 0.117 | 2.45 (1.02, 5.90) | | 0.046 |
| United Kingdom | | 1.82 (0.72, 4.58) | 0.204 | 1.06 (0.45, 2.49) | 0.891 | 0.83 (0.400, 1.71) | | 0.611 |
| United States | | 1.08 (0.59, 1.95) | 0.808 | 0.88 (0.48, 1.62) | 0.673 | 0.81 (0.47, 1.38) | | 0.435 |
| Caree condition | |  | 0.280 |  | 0.336 |  | | 0.324 |
| Alzheimer’s, Dementia | | 1 |  | 1 |  | 1 | |  |
| Cancer | | 0.95 (0.34, 2.72) | 0.931 | 0.88 (0.30, 2.55) | 0.807 | 2.32 (0.83, 6.50) | | 0.110 |
| Mental, emotional illness | | 1.44 (0.63, 3.29) | 0.390 | 0.84 (0.37, 1.91) | 0.676 | 1.43 (0.70, 2.92) | | 0.330 |
| Mobility, physical disability | | 1.12 (0.47, 2.66) | 0.800 | 1.71 (0.61, 4.77) | 0.307 | 0.80 (0.38, 1.71) | | 0.566 |
| “Old age”, frailty | | 0.45 (0.18, 1.11) | 0.083 | 1.50 (0.39, 5.77) | 0.555 | 0.80 (0.30, 1.71) | | 0.663 |
| Stroke | | 0.80 (0.38, 1.68) | 0.561 | 0.59 (0.28, 1.26) | 0.171 | 0.87 (0.46, 1.71) | | 0.721 |
